# Leptin alleviates endoplasmic reticulum stress induced by cerebral ischemia/reperfusion injury via the PI3K/Akt signaling pathway

**DOI:** 10.1042/BSR20221443

**Published:** 2022-12-12

**Authors:** Yan Zhang, Daobin Cheng, Chunxiao Jie, Tao Liu, Shixiong Huang, Shijun Hu

**Affiliations:** 1 Department of Rehabilitation, The Second Affiliated Hospital of Guangxi Medical University, Nanning, Guangxi 530007, People’s Republic of China; 2 Department of Neurology, The First Affiliated Hospital of Guangxi Medical University, Nanning, Guangxi 530021, People’s Republic of China; 3 Department of Neurology, Hainan General Hospital, Hainan Affiliated Hospital of Hainan Medical University, Haikou, Hainan 570311, People’s Republic of China

**Keywords:** cerebral ischemic/reperfusion injury, endoplasmic reticulum stress, leptin, PI3K/Akt signaling pathway

## Abstract

**Background:** Cerebral ischemic/reperfusion injury (CIRI) is a key factor for the prognosis of ischemic stroke (IS), the leading disease in terms of global disability and fatality rates. Recent studies have shown that endoplasmic reticulum stress (ERS) may be a target against CIRI and that leptin, a peptide hormone, has neuroprotective activity to mitigate CIRI.

**Methods**: An *in vitro* CIRI model was induced in primary cortical neurons by oxygen-glucose deprivation and reoxygenation (OGD/R) after pretreatment with LY294002 (10 µmol/L) and/or leptin (0.4 mg/L), and cell viability, neuronal morphology and endoplasmic reticulum (ER) dysfunction were evaluated. An *in vivo* CIRI model was established in rats by middle cerebral artery occlusion and reperfusion (MCAO/R) after the injection of LY294002 (10 μmol/L) and/or leptin (1 mg/kg), and neurological function, infarct volume, cerebral pathological changes, the expression of ERS-related proteins and cell apoptosis were examined.

**Results:** *In vitro*, leptin treatment improved the cell survival rate, ameliorated neuronal pathological morphology and alleviated OGD/R-induced ERS. *In vivo*, administration of leptin significantly reduced the infarct volume, neurological deficit scores and neuronal apoptosis as well as pathological alterations. In addition, leptin suppressed MCAO/R-induced ERS and may decrease apoptosis by inhibiting ERS-related death and caspase 3 activation. It also regulated expression of the antiapoptotic protein Bcl-2 and the proapoptotic protein Bax in the cortex. Furthermore, the inhibitory effect of leptin on ERS was significantly decreased by the effective phosphatidylinositol 3-kinase (PI3K) inhibitor LY294002.

**Conclusions:** These results confirm that ERS plays an important role in CIRI and that leptin can inhibit the activation of ERS through the PI3K/Akt pathway, thereby alleviating CIRI. These findings provide novel therapeutic targets for IS.

## Introduction

Stroke accounts for almost 5% of all disability-adjusted life years [1]. With enhanced aging of the population and changes in lifestyle, the incidence and mortality rate of stroke are increasing annually. Stroke mainly comprises two types, hemorrhagic stroke and ischemic stroke (IS). IS accounts for 71% of stroke events worldwide [2]. IS is characterized by interrupted cerebral blood flow, leading to insufficient energy supply, which initiates metabolic disorder and culminates in cerebral injury. Currently, the recanalization of vessels within a limited therapeutic window is a keg strategy in saving patients with IS [3]. However, reperfusion therapy may trigger complex pathological effects, such as oxidative stress, the inflammatory cascade and excessive apoptosis [4,5], a condition that is defined as cerebral ischemic/reperfusion injury (CIRI). Unfortunately, there are no effective treatments for CIRI to date.

The endoplasmic reticulum (ER), the organelle responsible for protein synthesis and folding, posttranslational modification and Ca^2+^ homeostasis, is sensitive to stress [6,7]. Under ischemic conditions, the abnormal accumulation of unfolded or misfolded proteins in the ER lumen triggers a pathological state called endoplasmic reticulum stress (ERS), which induces a cellular self-protective response termed the unfolded protein response (UPR) [8]. Recent studies have revealed that ERS is associated with enhanced damage from CIRI [9–11], and targeting ERS may be a novel therapeutic strategy for IS.

Obesity is well known to be a crucial risk factor for IS in all racial and ethnic groups and significantly increases the risk of atherosclerosis, which is a vital pathological mechanism involved in IS [12,13]. Leptin, one of the most common adipocytokines, is encoded by the obese gene and principally synthesized and secreted by adipocytes. Leptin not only plays a decisive role in metabolic homeostasis but also is involved in myriad pathophysiological processes, including inflammation regulation, proliferation and apoptosis [14–16]. An accumulating number of studies have demonstrated that exogenous leptin can bind its receptors in cerebral tissue after crossing the blood‒brain barrier and exert an antiapoptotic effect in CIRI [17–19]. The neuroprotective mechanism of leptin involves multiple antiapoptotic pathways, including the phosphatidylinositol 3-kinase (PI3K)/Akt signaling pathway [17]. Akt, also known as protein kinase B, is an important survival factor related to brain development, aging and disease and is involved in a myriad of cellular processes, such as glucose metabolism, protein synthesis and ERS [20–22]. However, the neuroprotective properties of leptin in alleviating ERS remain unclear.

In this context, we aimed to explore the effects of leptin on intracellular ERS and ERS-associated apoptosis in CIRI. Moreover, we also focused on the link between ERS induced by CIRI and the PI3K/Akt signaling pathway.

## Materials and methods

### Animals and ethics

All animal experiments in our study took place at the Experimental Animal Center of Guangxi Medical University. Adult male Sprague‒Dawley rats (250–280 g) and newborn SD rats (within 24 h) were supplied by the Experimental Animal Center of Guangxi Medical University. All experimental procedures involving animals were performed following an ethical review of animal welfare administered by The Animal Care & Welfare Committee of Guangxi Medical University (project proposal number 2020006014).

### Preparation of rat primary neurons

Cortical neurons were extracted from newborn SD rats. Briefly, 1-day-old newborn rats were euthanized with excessive phenobarbital immediately before neuron extraction. Then, the dissected cortical tissues were sheared separately into small fragments and digested in 0.25% trypsin for 10 min, and DMEM/F-12 containing 10% fetal bovine serum was added for neutralization. The neurons were centrifuged for 5 min at 1000 rpm at 4°C and subsequently resuspended in DMEM/F-12, followed by plating in poly-D-lysine-precoated plates. After 4 h, the original medium was replaced with neurobasal-A medium supplemented with 2% B27 and 1% GlutaMAX. The purity of the neurons was identified based on the marker NeuN on the 7th day.

### Leptin pretreatment and OGD/R modeling

For this study, recombinant rat leptin (Roche Pharmaceuticals, Switzerland), at doses of 0, 0.1, 0.2, 0.4 and 0.8 mg/L, was used to pretreat the neurons for 24 h, followed by OGD/R. Furthermore, LY294002 (MedChemExpress, U.S.A.), an inhibitory of PI3K, at a dose of 10 μmol/L was applied 30 min before intervention with leptin in the LY+leptin group. DMSO (1%) was used to treat the neurons in the vehicle group.

The OGD/R model was established on the 7th day of neuronal culture. In brief, the neurobasal-A medium was removed and replaced with EBSS without glucose. Later, the neurons were placed in a modular incubator filled with 5% CO_2_/95% N_2_ for 1, 2 or 4 h. After that, the EBSS was replaced with neurobasal-A medium, and then the neurons were cultured for another 24 h in a normal incubator filled with 5% CO_2_ at 37°C. The medium for neurons in the control group was replaced with new medium, and these neurons were not subjected to OGD/R treatment.

### Cell viability assay

The Cell Counting Kit-8 (CCK-8) assay was used to assess neuron viability. The neurons were plated in 96-well plates at 2 × 10^5^ cells/cm^2^. The blank group consisted of an equal volume of medium without cells. After reoxygenation for 24 h, 10 μl of CCK-8 solution was applied to each well and incubated with the cells for 3 h in the dark at 37°C. The absorbance at 450 nm was detected with an spectrophotometer (Thermo Scientific, U.S.A.). The cell viability was calculated as follows: cell viability (%) = (experimental group − blank group)/(control group -- blank group)×100%.

### Experimental groups and middle cerebral artery occlusion and reperfusion (MCAO/R)

After 3 days of environmental adaptation, 80 male SD rats were randomly divided into four groups according to the ARRIVE guidelines. Rats in the sham group underwent the same operation without monofilament insertion. Rats in the vehicle group were subjected to MCAO/R and given 0.9% saline after MCAO. As described by Zhang et al. [23], the rats in the leptin group were immediately given recombinant rat leptin at a dose of 1 mg/kg after MCAO surgery. The rats in the LY+leptin group, in addition to leptin administration, was pretreated with LY294002 (10 μmol/L via the tail vein) 30 min before surgery. A total of 0.5 ml of 1% DMSO was given by tail vein injection to rats in the vehicle group and the leptin group before surgery as controls for LY294002.

Prior to the surgery, the rats were fasted overnight but given sterile water. The rats were anesthetized with 40 mg/kg pentobarbital administered intraperitoneally. The surgical region was shaved and sterilized with iodophor. Surgery was performed on an animal operating table with an insulated pad. As previously described [24,25], the right common carotid artery (CCA), external carotid artery (ECA) and internal carotid artery (ICA) were identified through a midline neck incision and isolated with the vagus nerve and carotid bifurcations. The right CCA near the heart and ECA were ligated, and an oblique incision, approximately 5 mm from the right ICA, was cut at the right CCA. An intraluminal monofilament was introduced into the ICA at approximately 18 mm. After 2 h, the intraluminal monofilament was retracted. The incision was sutured in layers and disinfected with iodophor. The rectal temperature of the rats remained at 37.0 ± 0.5°C, and the respiratory rhythm of the rats remained stable during surgery. Rats with no neurologic deficits and rats that died were excluded from the experiment. After that, the rats were placed in cages, where the animals could freely consume food and water, and reperfusion was continued for 24 h. The researchers who carried out subsequent experiments were blinded to these groups.

### Evaluation of neurologic deficits

As described previously [24], Longa’s 5-point scale was used to evaluate neurologic function with the following scoring system: 0 = no neurologic deficit; 1 = paralysis and inability to fully extend the anterior or hind limbs; 2 = circling to the paralyzed side; 3 = falling to the paralyzed side and crawling; and 4 = unable spontaneously to walk and loss of consciousness.

### Measurement of the infarct area

The cerebral infarct volume was measured after neurological examination. Briefly, the rats were anesthetized deeply with 4% pentobarbital, and the brains were rapidly dissected out and then refrigerated for 20 min. After that, coronal segments at a thickness of 3 mm were cut. Then, the segments were incubated with a 2% 2,3,5-triphenyltetrazolium chloride (TTC) solution for 20 min at 37°C. ImageJ software was used to analyze the total infarct area of each segment, and the cerebral infarct volume data are presented as follows: the percentage of [(contralateral volume) − (ipsilateral undamaged volume)]/contralateral volume × 100% [26].

### Histopathological evaluation

Hematoxylin–eosin staining was used for histological evaluation. After perfusion with 0.9% saline, the brains of the rats were isolated and fixed with a 4% paraformaldehyde solution overnight at 4°C, after which they were embedded in paraffin. Afterward, coronal sections at a thickness of 5 µm were cut and frozen at −20°C for preservation. The prepared sections were soaked in xylene followed by gradient ethyl alcohol and then stained with hematoxylin and eosin. Then, the pathological morphology of the cerebral cortex was observed under a fluorescence microscope (OLYMPUS, Japan).

### TUNEL staining

The prepared sections were dewaxed with xylene and then washed in an alcohol gradient. After incubation with a 0.25% Triton X-100 solution for 20 min, the sections were covered with a prepared TUNEL reaction mixture (Roche Pharmaceuticals, Switzerland) and incubated for 1 h in a dark room. Finally, each section was mounted with fluorescence decay-resistant medium and visualized under a fluorescence microscope. The data are presented as the apoptotic index (apoptotic index = the number of positive cells in each field/all cells in the field-× 100%).

### Transmission electron microscopy

Morphological alteration of the endoplasmic reticulum was measured by using transmission electron microscopy (TEM). Briefly [27], primary neurons were fixed with 2.5% glutaraldehyde overnight and then embedded with 100% epoxy resin. Furthermore, ultrathin sections of 70–80 nm thickness were cut using an ultra microtome. Subsequently, sections were stained with 2% uranyl acetate and 2% lead acetate. Finally, the sections were observed under a transmission electron microscope (GOM, Germany) and the morphology of ER were observed.

### Double immunofluorescence staining

A 4% paraformaldehyde solution was used to fix sections of the primary neurons or brain tissues for 30 min. Then, the sections were incubated with a 0.25% Triton X-100 solution for 15 min at room temperature. After blocking with 10% goat serum, the sections were incubated with the following primary antibodies at 4°C: anti-p-Akt (ab38449, 1:200, Abcam, U.S.A.), anti-GRP78 (ab21685, 1:250, Abcam, U.S.A.), anti-CHOP (#5554, 1:200, Cell Signaling Technology, U.S.A.) and anti-NeuN (GB11138, 1:200, Servicebio Technology, China). After overnight incubation, the sections were incubated with anti-mouse secondary antibodies (A48255,1:200, Thermo Scientific, U.S.A.) and anti-rabbit secondary antibodies (PA1-28786, 1:200, Thermo Scientific, U.S.A.) for 1 h. Finally, the sections were mounted with fluorescence decay-resistant medium and visualized under a fluorescence microscope.

### Western blot analysis

Primary cortical neurons or brain tissues were lysed with RIPA buffer containing phosphatase and protease inhibitors. After measurement with BCA kits, the proteins were denatured by boiling for 10 min and then loaded on 8-12% SDS‒PAGE gels, followed by transfer from the gels to activated PVDF membranes. After blocking with 5% nonfat dry milk, the membranes were incubated with the following primary antibodies: anti-PI3K (ab154598, 1:2000), anti-p-Akt (ab38449, 1:1000), anti-Akt (ab283852, 1:2500), anti-GRP78 (ab21685, 1:3000), anti-p-PERK (ab192591, 1:1000), anti-PERK (ab79483, 1:2500), and anti-p-IRE1α (ab124945, 1:2500) were obtained from Abcam, USA and anti-IRE1α (#3294, 1:2500), anti-ATF6 (#65880, 1:2000), anti-CHOP (#5554, 1:2000), anti-Caspase12 (#35965, 1:2500), anti-Caspase3 (#9579, 1:1000), anti-Bcl-2 (#3869S, 1:2000) and anti-Bax (#5023, 1:2500) were supplied by Cell Signaling Technology, U.S.A. Anti-β-actin antibody (GB15004, 1:3000) was purchased from Servicebio Technology (China). After incubation with the primary antibodies overnight, the membranes were washed three times with TBST and incubated with secondary anti-rabbit HRP antibody (GB1213, 1:5000, Servicebio Technology, China). Enhanced chemiluminescence was used to visualize the membranes, and the analysis was performed using ImageJ software.

### Statistical analyses

All statistical analyses were performed using SPSS 22.0 software, and the data are shown as the means ± SEMs. *In vivo* experiments were repeated three times, and *in vitro* experimental results are representative of at least three independent experiments. Shapiro–Wilk test was performed to ensure normality of the data. One-way analysis of variance was used to analyze measurement data for multiple comparisons, including data from Western blotting, double immunofluorescence staining, TUNEL staining, calculation of the infarct area, cell viability assays and RT-PCR. The differences between the two groups were detected with LSD post hoc test. Nonparametric Kruskal–Wallis test followed by a Bonferroni-corrected Mann–Whitney *U-*test were conducted for skewed data, specifically that obtained by evaluation of neurologic deficits. Differences for which *P*<0.05 were considered statistically significant.

## Results

### Identification of neurons, determination of the OGD time and confirmation of the leptin concentration

**Figure 1:**
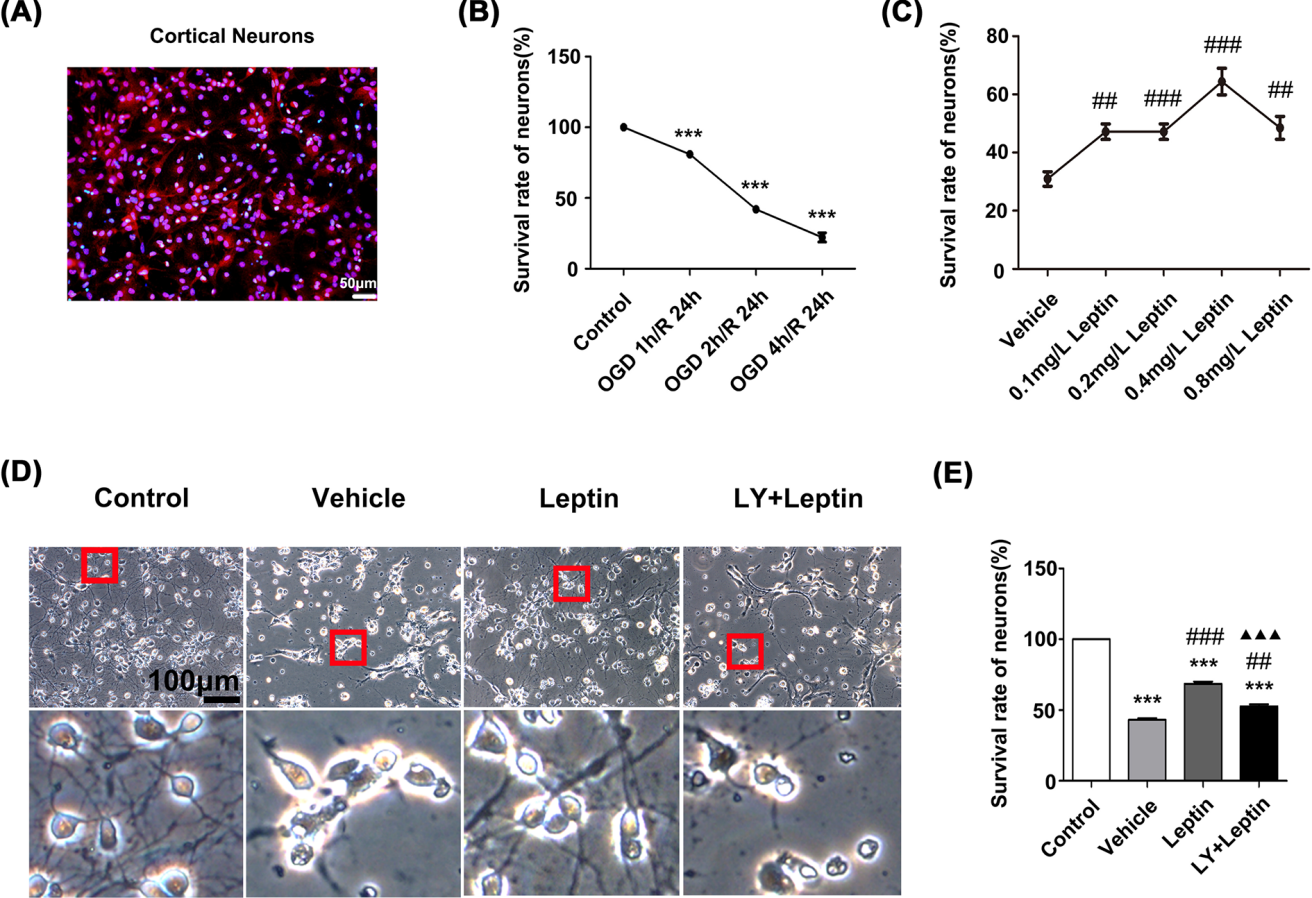
Leptin protected primary cortical neurons against OGD/R injury (**A**) Cortical neurons were labeled with NeuN and DAPI. (**B**) The viability of neurons that were subjected to OGD for different degrees (0, 1, 2, 4 h) followed by reoxygenation for 24 h. (**C**) The viability of neurons that were treated with different concentrations of leptin (0, 0.1, 0.2, 0.4 or 0.8 mg/L) and subjected to OGD for 2 h/R for 24 h. (**D**) Representative images of neurons in each group under a microscope (scale bar: 100 μm). (**E**) The viability of cortical neurons in each group. The data are presented as the means ± SEMs (*n*=3). ***P<0.001, versus the control group; ^##^*P*<0.01, versus the vehicle group; ^###^*P*<0.001, versus the vehicle group; ^▲▲▲^*P*<0.001, versus the leptin group.

Immunofluorescence staining was used to ensure the purity of the neurons to ensure the accuracy of the *in vitro* experiments. All nuclei were stained blue with DAPI, and neuronal nuclei were stained red with NeuN. The neurons accounted for more than 90% of the cells (Figure 1A).

To select the optimal time to establish the OGD model, neurons were deprived of glucose and oxygen for 1, 2 and 4 h and then reoxygenated for 24 h. After that, the viability of the neurons at different time points was determined by a CCK-8 assay. Cell viability gradually decreased as the OGD time increased. After OGD for 2 h and reoxygenation for 24 h, the viability of the cells dramatically decreased to 42.06%. Considering the injury process during reperfusion, cells were subjected to OGD for 2 h and reoxygenation for 24 h to model CIRI *in vitro* (Figure 1B).

To study the neuroprotective effect of leptin, neurons were pretreated with leptin at doses of 0.1, 0.2, 0.4 and 0.8 mg/L. Afterward, the viability of the cells was examined using a CCK-8 assay. The results showed that cell viability with the addition of 0.4 mg/L leptin was significantly higher than that in the vehicle group (*P*<0.001). After comprehensive evaluation, the optimal concentration of leptin was determined to be 0.4 mg/L (Figure 1C).

### Leptin improves cell morphology and viability

Normal neurons from the control group had clearly defined, round and bright nuclei with abundant cytoplasm and integrated neuronal networks. After OGD/R, the neurons from the vehicle group shrank, their cytoplasm was reduced in volume, and the neuronal networks gradually disappeared (Figure 1D). However, after treatment with LY294002, an inhibitor of PI3K, the neuroprotective effect of leptin was weakened in the LY+leptin group, as demonstrated by increased damage to the cell morphology and a failure to improve cell viability (Figure 1D,E).

Cell morphology was observed by a phase-contrast inverted microscope. Normal neurons from the control group had clearly defined, round and bright nuclei with abundant cytoplasm and integrated neuronal networks. After OGD/R, the neurons from the vehicle group shrank, their cytoplasm was reduced in volume, and the neuronal networks gradually disappeared (Figure 1D). Compared with the vehicle group, the leptin group exhibited fewer morphological alterations. Furthermore, the results of the CCK-8 assay were consistent with the results of microscopic observation, as the CCK-8 assay showed that the cell viability in the leptin group was significantly improved in comparison to that in the vehicle group. However, after treatment with LY294002, an inhibitor of PI3K, the neuroprotective effect of leptin was weakened in the LY+leptin group, as demonstrated by increased damage to the cell morphology and a failure to improve cell viability (Figure 1D,E).

### Leptin activates the PI3K/Akt signaling pathway during OGD/R

**Figure 2:**
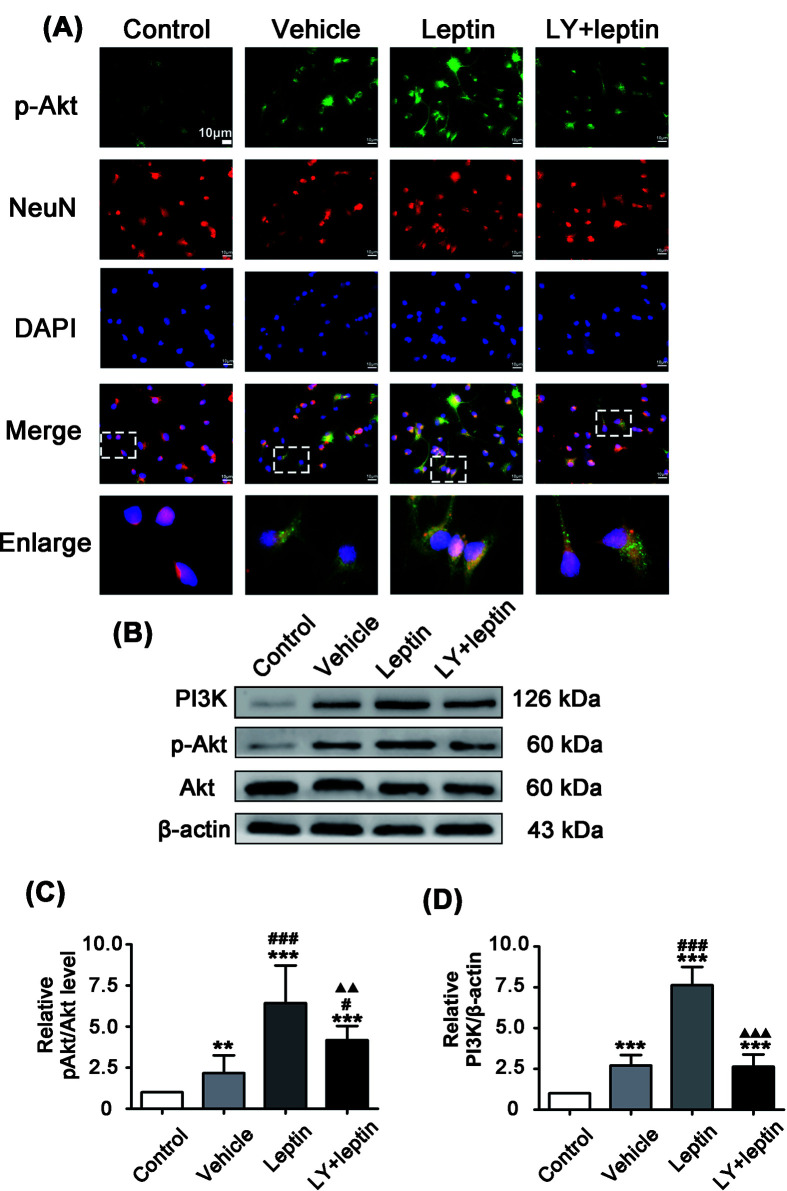
Leptin pretreatment up-regulated p-Akt expression during OGD/R (**A**) Cortical neurons costained for p-Akt (green), NeuN(red) and DAPI (blue) (scale: 10 μm). (**B–D**) Representative western blot images and quantitation of PI3K and p-Akt. The quantitative results are presented as the mean ± SEM (*n* = 3 per group). **P<0.01, versus the control group; ***P<0.001, versus the control group; *^#^P*<0.05, versus the vehicle group; ^###^*P*<0.001, versus the vehicle group; ^▲▲^*P*<0.01, versus the leptin group; ^▲▲▲^*P*<0.001, versus the leptin group.

To explore the effect of the PI3K/Akt signaling pathway on leptin-mediated neuroprotection during OGD/R, we examined the level of Akt phosphorylation in neurons. The results of both immunofluorescence staining (Figure 2A) and Western blotting (Figure 2B–D) showed that PI3K and p-Akt were significantly increased in the vehicle group in comparison with their levels in the control group. With leptin pretreatment, PI3K and p-Akt expression was further increased in the leptin group compared with the vehicle group. After pretreatment with LY294002, we found a significant decrease in PI3K and p-Akt expression in the LY+leptin group compared with the leptin group (Figure 2B–D), indicating that leptin activated the PI3K/Akt pathway *in vitro*.

### Leptin inhibits OGD/R-induced ERS via the PI3K/Akt signaling pathway

**Figure 3:**
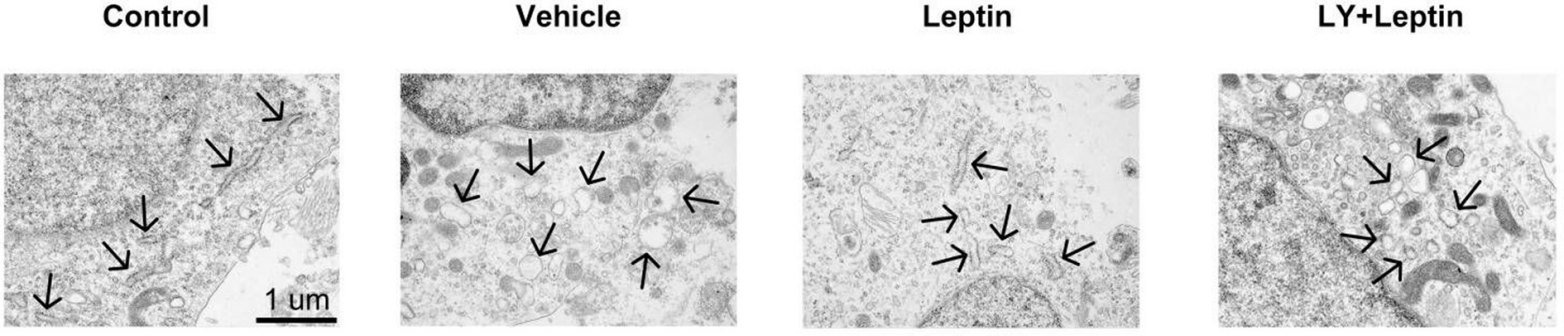
Leptin ameliorated neuronal ER damaged induced by OGD/R Endoplasmic reticulum morphological changes were observed by transmission electron microscopy (scale bars: 1 μm). The black arrows point to the ER structure.

To study whether leptin alleviates OGD/R-induced ERS, ER structure and expression were observed. In TEM analysis, neurons in the control group were observed to have normal cell morphology with complete cell membranes, nuclear membranes, and normal ER structure which manifested as a flattened and interconnected tubular network surrounded by massive ribosomes. In the vehicle group, the ER was dilated and partly fragmented with fewer studded ribosomes and shorter length. The pathological alterations in ER structure were greatly prevented by leptin pretreatment as reflected in leptin group, whereas LY294002 pre-treatment interfered with the protective effect of leptin on ER structure (Figure 3).

**Figure 4:**
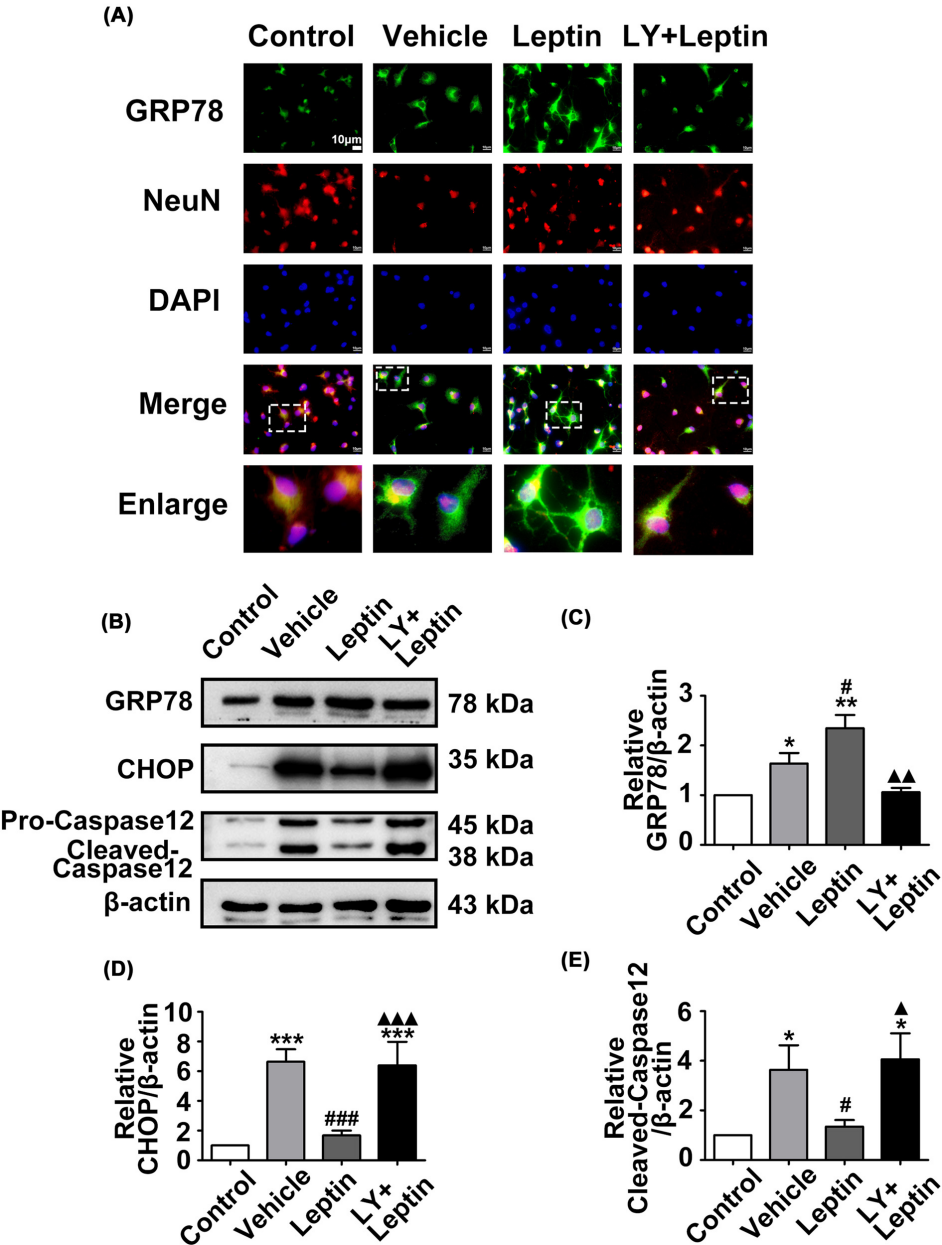
Leptin pretreatment increased GRP78 expression and decreased Caspase12 and CHOP levels after OGD/R (**A**) Cortical neurons costained for GRP78 (green), NeuN(red) and DAPI (blue) (scale: 10 μm). (**B–E**) The relative western blot images and analysis of GRP78, Caspase12 and CHOP in neurons in each group. The quantitative results are presented as the mean ± SEM (*n* = 3 per group). *P<0.05, versus the control group; **P<0.01, versus the control group; ***P<0.001, versus the control group; ^#^*P*<0.05, versus the vehicle group; ^#^*P*<0.05, versus the vehicle group; ^###^*P*<0.001, versus the vehicle group; ^▲^*P*<0.05, versus the leptin group; ^▲▲^*P*<0.01, versus the leptin group; ^▲▲▲^*P*<0.001, versus the leptin group.

Furthermore, we investigated the impact of leptin on the expression of ERS markers. The immunofluorescence staining revealed that Akt was phosphorylated after subjected to OGD/R (Figure 4A). The Western blot results showed that GRP78, CHOP and Caspase12 levels were significantly increased in the vehicle group compared with the control group. Leptin pretreatment significantly reduced expression of the proapoptotic proteins CHOP and Caspase12 and further enhanced expression of the prosurvival protein GRP78 in the leptin group compared with the vehicle group. However, the LY+leptin group showed an increase in Caspase12 and CHOP expression and a decrease in the expression of GRP78 in comparison with their expression in the leptin group (Figure 4B–E).

### Leptin exerts a neuroprotective effect against MCAO/R injury

**Figure 5:**
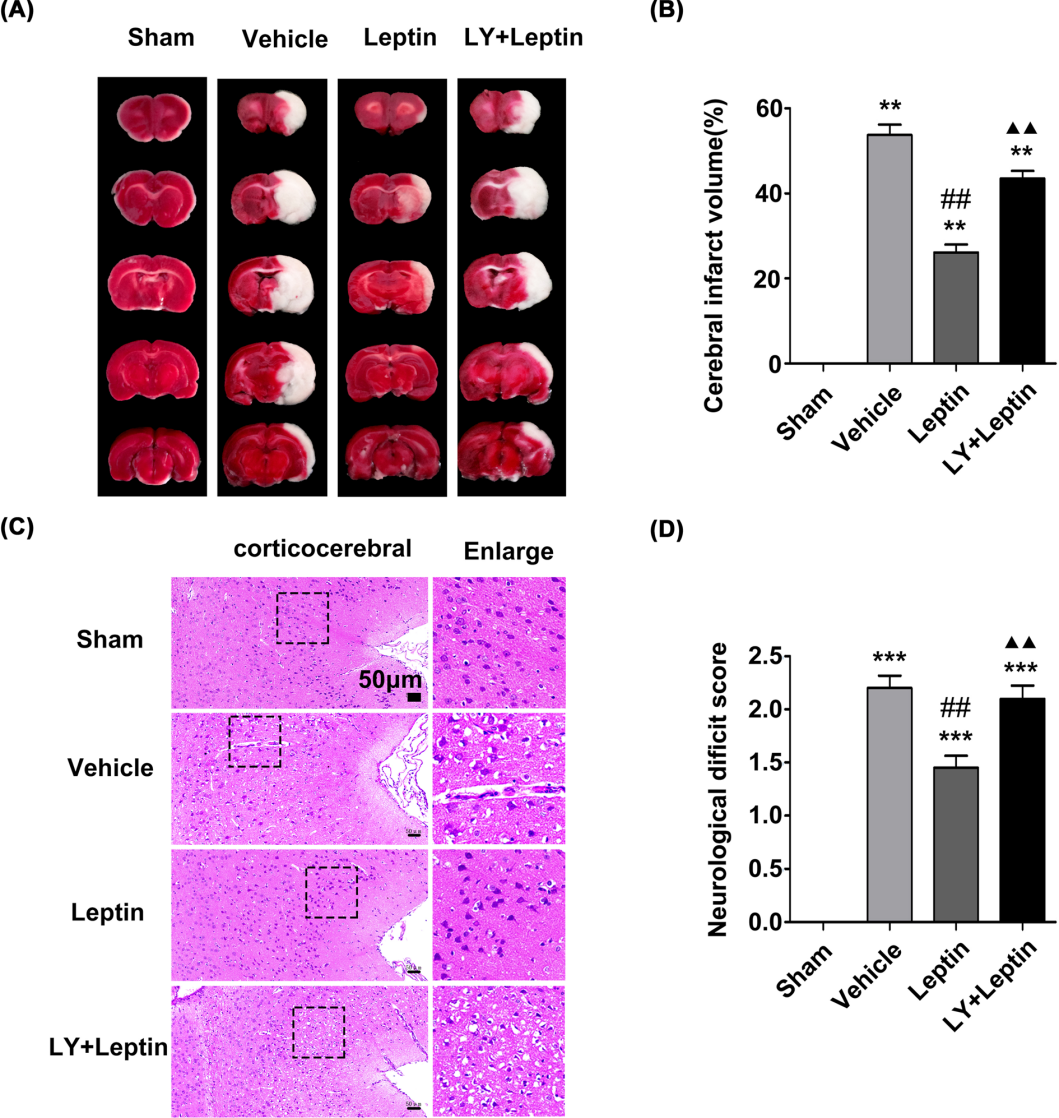
Leptin pretreatment decreased the infarct volume, alleviated pathological changes and reduced neurological deficit scores (**A,B**) Representative images of TTC staining and the corresponding proportion of infarct area at 24 h after reperfusion. (**C**) Representative images of HE staining in the cerebral ischemic area (scale: 50 μm). (**D**) The neurological deficit score in each group of rats after 24 h of reperfusion. The quantitative results are presented as the mean ± SEM (*n* = 4 per group). **P<0.01, versus the control group; ***P<0.001, versus the control group; ^##^*P*<0.01, versus the vehicle group; ^▲▲^*P*<0.01, versus the leptin group.

TTC staining showed that compared with that in the sham group, an increased infarct volume (white area) was observed in the vehicle group. The infarct lesions were significantly decreased in volume in the leptin group compared with the vehicle group, and this effect of leptin was reversed by LY294002 (Figure 5A,B).

As indicated by HE staining, rats in the sham group showed a normal cortical structure, round or cone-shaped nerve cells with obvious nuclei, continuous cell membranes and rich cytoplasm. Rats in the vehicle group showed a loose tissue structure, irregular nerve cells with solidified and fragmented nuclei, and a large number of cytoplasmic vacuoles. Damage to the cerebral cortex in the leptin group was reduced to varying degrees compared with that in the vehicle group, and other pathological changes were also alleviated. However, after pretreatment with LY294002, the neurons showed nuclear pyknosis and cleavage. Neuronal loss and cerebral edema were evident (Figure 5C).

After MCAO surgery, the neurological deficit score was evaluated after 24 h of reperfusion to investigate the neuroprotective efficacy of leptin. No neurological deficit symptoms were observed in the sham group, while rats presented neurological deficits to varying degrees after MCAO/R. The neurological deficit score in the leptin group was lower than that in the vehicle group. However, the neurological deficit score in the LY+leptin group was significantly higher than that in the leptin group (Figure 5D).

### Leptin activates the PI3K/Akt signaling pathway during MCAO/R

**Figure 6:**
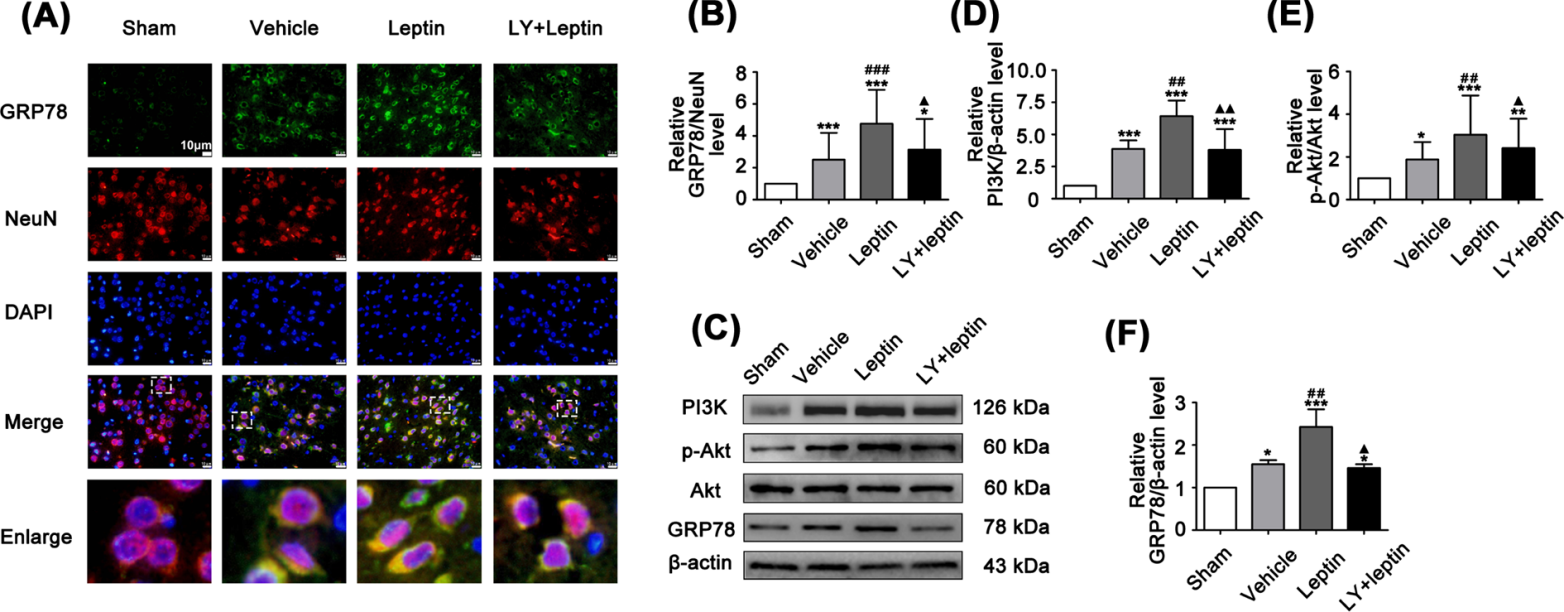
Leptin upregulated p-Akt and GRP78 expression in MCAO/R rats (**A,B**) Semiquantitative analysis of GRP78 expression and costaining for GRP78 (green), NeuN (red) and DAPI (blue) in the ischemic cortex (scale: 10 μm). (**C–F**) Representative western blot images and quantitation of PI3K, p-Akt and GRP78. The quantitative results are presented as the mean ± SEM (*n* = 3 per group). *P<0.05, versus the control group; **P<0.01, versus the control group; ***P<0.001, versus the control group; ^#^*P*<0.05, versus the vehicle group; ^##^*P*<0.01, versus the vehicle group; ^###^*P*<0.001, versus the vehicle group; ^▲^*P*<0.05, versus the leptin group; ^▲▲^*P*<0.01, versus the leptin group.

LY294002 was applied to explore whether the PI3K/Akt signaling pathway was related to the suppressive effect of leptin on ERS *in vivo*. Consistent with the in vitro results, the expression of PI3K and p-Akt was significantly increased in the leptin group compared with the vehicle group. Nevertheless, these effects on PI3K and p-Akt became eased in the LY+leptin group compared with the leptin group (Figure 6D,E).

### Leptin inhibits MCAO/R-induced ERS via the PI3K/Akt signaling pathway

**Figure 7:**
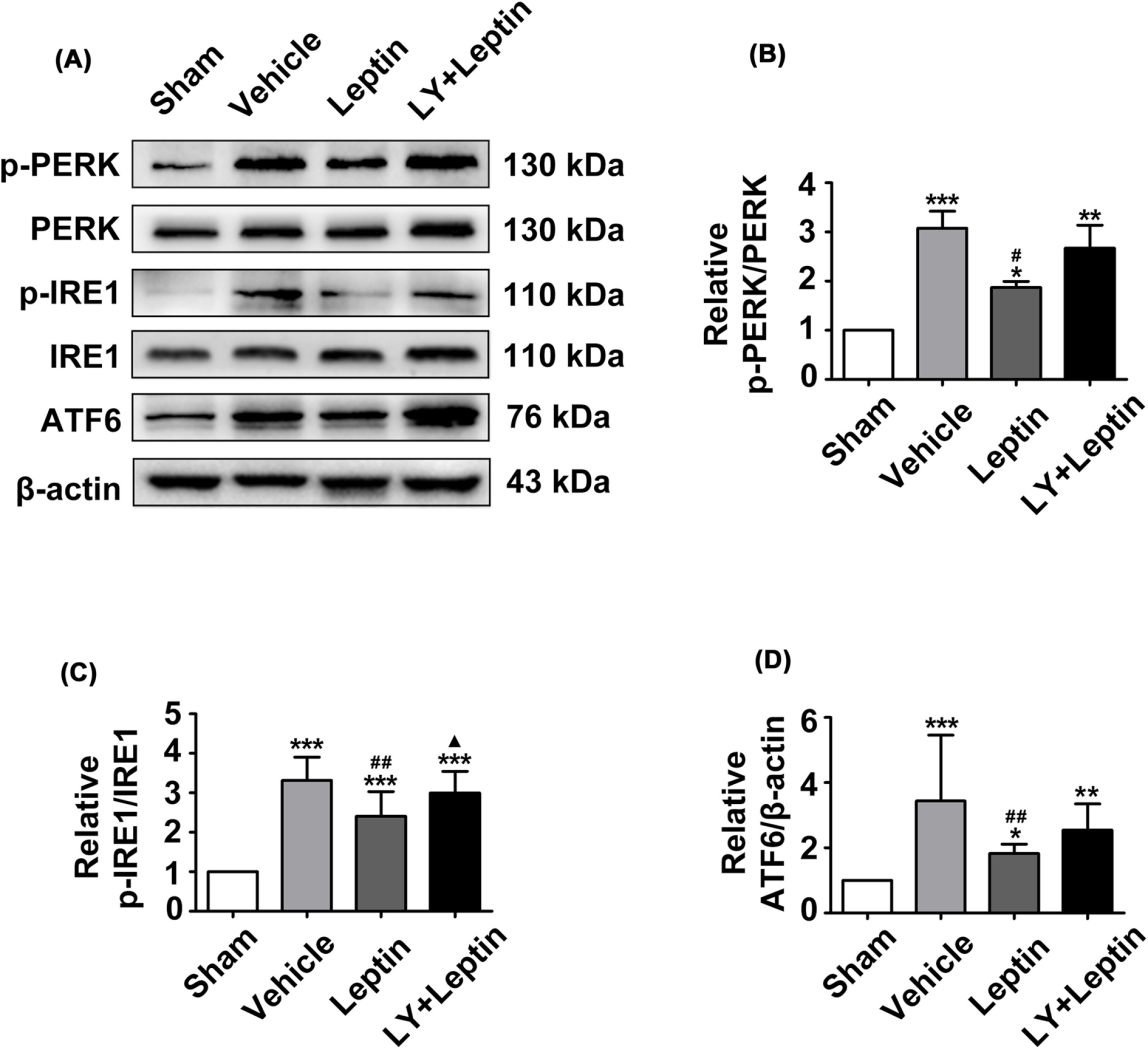
Leptin down-regulated the protein levels of p-PERK, p-IRE1 and ATF6 (**A-D**) Representative western blot images and quantitation of p-PERK, p-IRE1 and ATF6. The quantitative results are presented as the mean ± SEM (*n* = 3 per group). *P<0.05, versus the control group; **P<0.01, versus the control group; ***P<0.001, versus the control group; ^#^*P*<0.05, versus the vehicle group; ^##^*P*<0.01, versus the vehicle group; ^▲^*P*<0.05, versus the leptin group.

**Figure 8:**
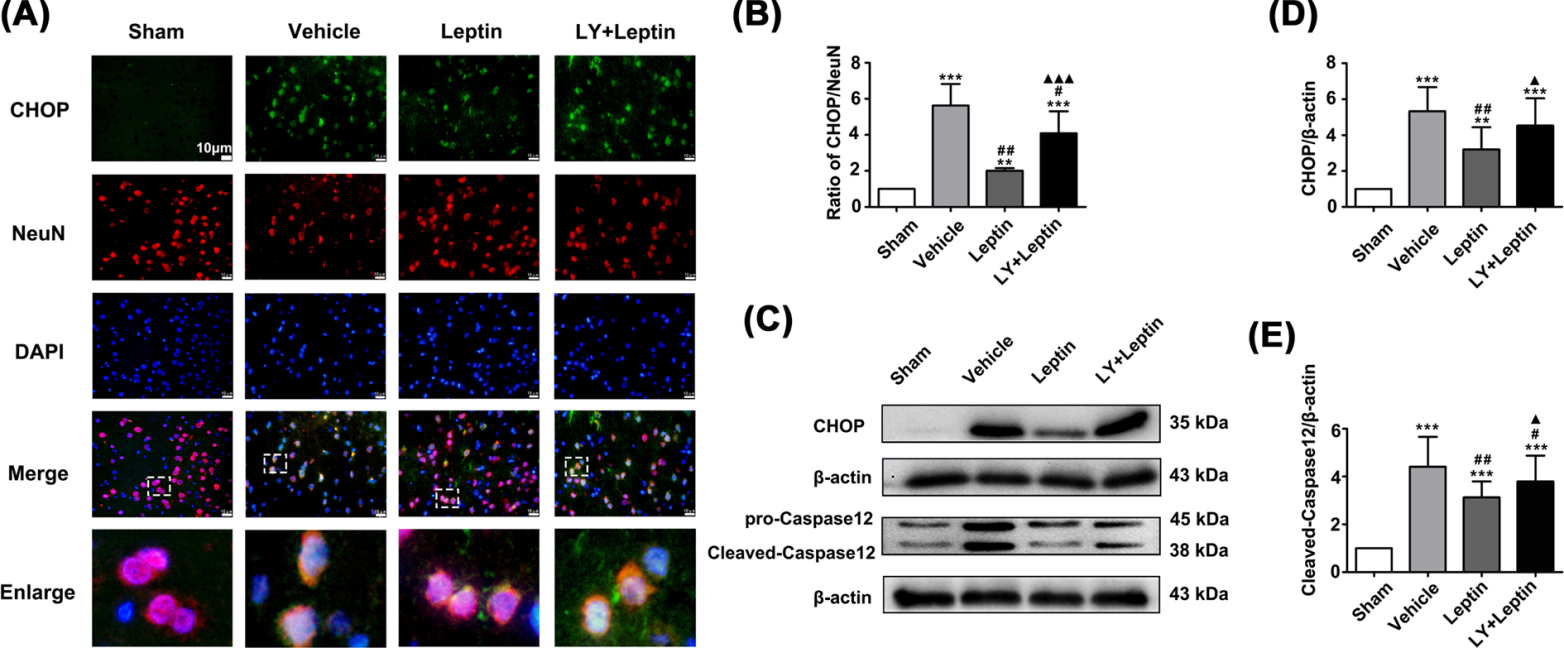
Leptin decreased the expression of CHOP and Caspase12 (**A,B**) Costained for CHOP (green), NeuN(red) and DAPI (blue) (scale bar: 10 μm) and semiquantitative analysis of CHOP expression in the ischemic cortex. (**C-E**) Representative western blot images and quantitation of CHOP and Caspase12. The quantitative results are presented as the mean±SEM (n = 3 per group). **P<0.01, versus the control group; ***P<0.001, versus the control group; ^#^*P*<0.05, versus the vehicle group; ^##^*P*<0.01, versus the vehicle group; ^ ▲^*P*<0.05, versus the leptin group; ^▲▲▲^*P*<0.001, versus the leptin group.

The results of immunofluorescence staining and Western blotting showed that MCAO/R significantly regulates the occurrence of ERS, as demonstrated by an increase in the expression of GRP78, p-PERK/PERK, p-IRE1/IRE1, ATF6, Caspase12 and CHOP in the vehicle group compared to the sham group (Figures 6-8). The leptin group showed considerable decreases in the levels of p-PERK/PERK, p-IRE1/IRE1, ATF6, Caspase12 and CHOP, whereas a further increase in GRP78 expression in comparison to that in the vehicle group was observed (Figures 6-8). However, LY294002 pretreatment significantly aggravated ERS, as shown in the LY+leptin group. The levels of the ERS-related proteins Caspase12 and CHOP (Figure 8) and the levels of IRE1 and PERK phosphorylation were increased (Figure 7), while the expression of GRP78 was decreased significantly compared with that in the leptin group (Figure 6A,B). These results demonstrated that blocking the PI3K/Akt signaling pathway affected the inhibitory effect of leptin on ERS.

### Leptin reduced the apoptotic rate and apoptosis-related protein levels in vivo

**Figure 9:**
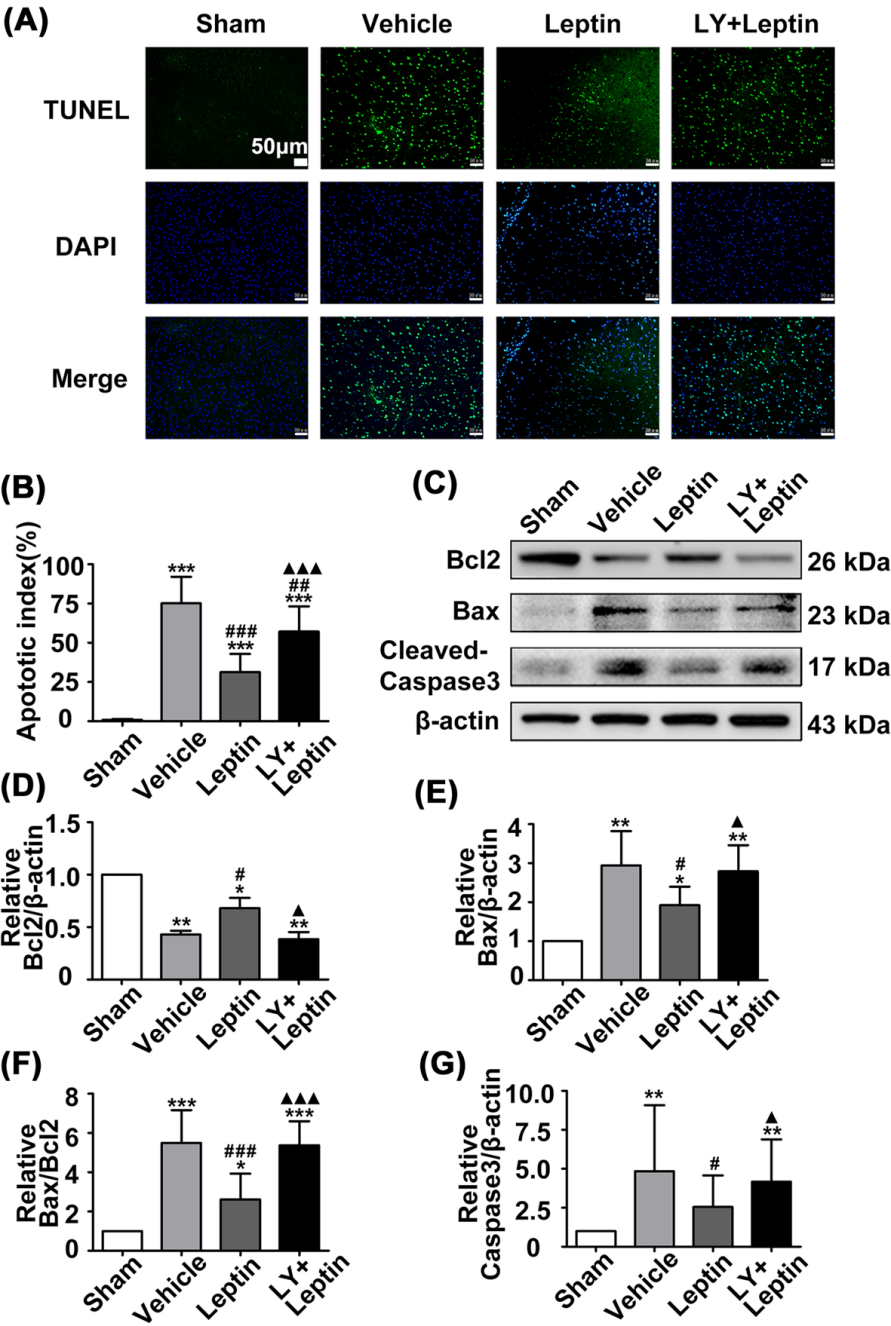
Leptin reduced the apoptotic rate, increased Bcl2 expression, and decreased Bax and cleaved-Caspase3 expression (**A,B**) TUNEL staining in ischemic cortical area (scale: 20 μm) and quantification of TUNEL-positive neurons. (**C–G**) Representative western blot images and quantitation of Bcl2, Bax and cleavedCaspase3. The quantitative results are presented as the mean ± SEM (*n* = 3 per group). *P<0.05, versus the control group; **P<0.01, versus the control group; ***P<0.001, versus the control group; ^#^*P*<0.05, versus the vehicle group; ^##^*P*<0.01, versus the vehicle group; ^###^*P*<0.001, versus the vehicle group; ^▲^*P*<0.05, versus the leptin group; ^▲▲▲^*P*<0.001, versus the leptin group.

TUNEL staining was performed to assess neuronal apoptosis in the cerebral cortex of the rats. In contrast to that in the sham group, the number of TUNEL-positive cells was significantly higher in the vehicle group. Interestingly, leptin treatment obviously decreased the rate of neuronal apoptosis in the leptin group in comparison to the vehicle group. However, the LY+leptin group showed a distinct increase in the percentage of TUNEL-positive cells when compared with that of the leptin group (Figure 9A,B).

**Figure 10:**
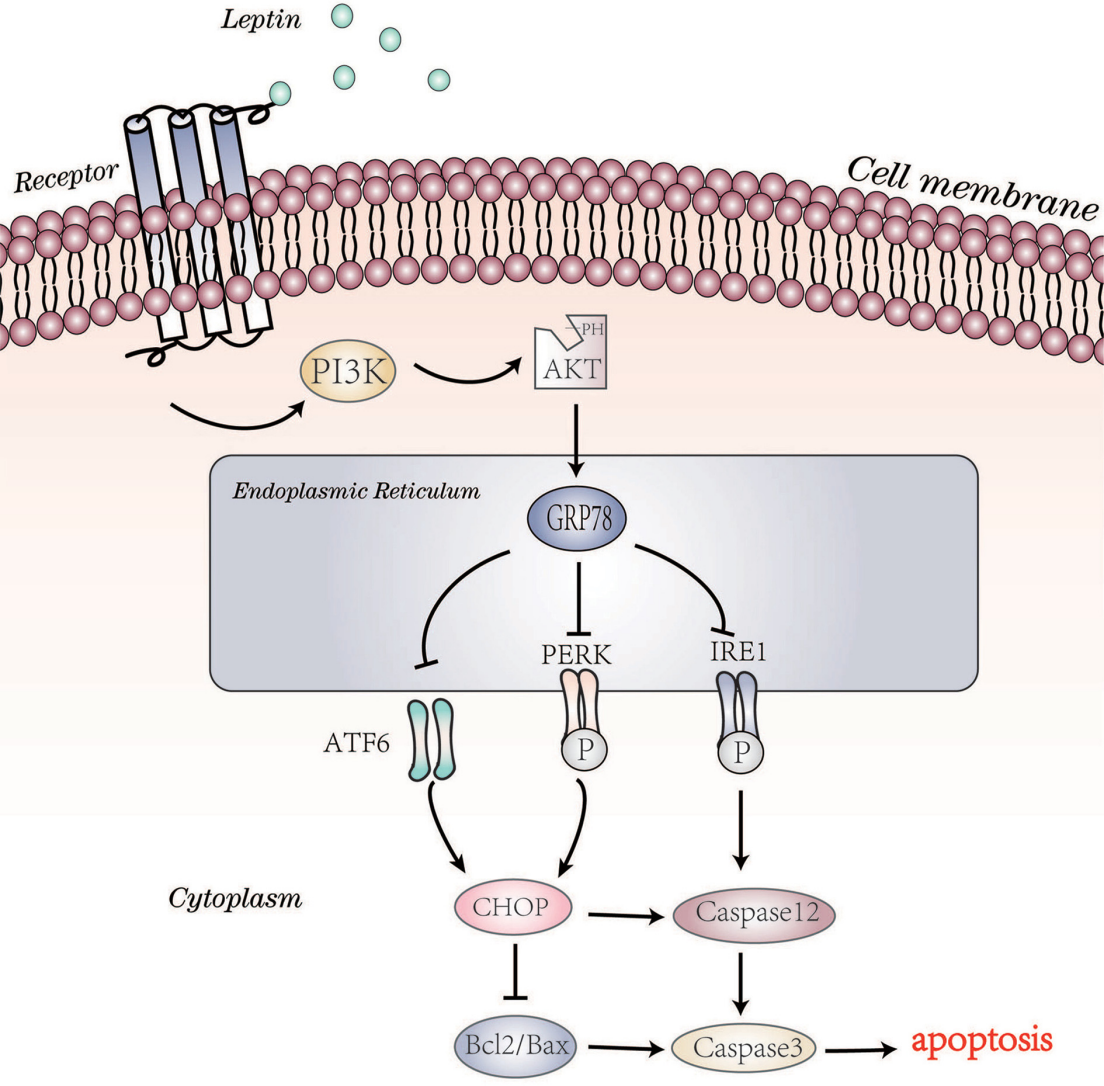
Molecular pathways involved in the neuroprotective effect of leptin against CIRI

The deterioration of ERS can result in apoptosis. In this study, expression of the proapoptotic protein Bax and the antiapoptotic proteins Bcl2 and Caspase3 was examined. In the vehicle group, the expression of Bax and cleaved Caspase3 was increased, while the expression of Bcl2 was decreased compared with that in the sham group. Leptin treatment reduced the Bax/Bcl2 ratio and cleaved Caspase3 expression levels compared with those in the vehicle group. However, LY294002 pretreatment significantly decreased the expression of Bax, Bcl2 and cleaved Caspase3 in the LY+leptin group (Figure 9C–G), suggesting that leptin alleviates ERS-mediated apoptosis through the PI3K/Akt signaling pathway (Figure 10).

## Discussion

In the present study, a primary neuronal injury model of OGD/R and a rodent surgical model of MCAO/R were established to mimic the clinical features of CIRI *in vitro* and *in vivo*, respectively. Our results showed that *in vitro*, pretreatment with leptin improved the survival rate of neurons and maintained neuronal morphology; *in vivo*, the administration of leptin reduced the cerebral infarct volume, morphological damage and neuronal apoptosis, revealing that leptin reduces secondary damage due to brain injury after ischemia. Furthermore, we found that leptin treatment significantly alleviated CIRI-induced ultrastructural damage to the ER and reduced the expression of ERS-related proteins and proapoptotic factors, indicating that leptin may inhibit ERS and ERS-induced death to exert its neuroprotective efficacy. In addition, we explored the potential mechanism of leptin by using a PI3K inhibitor and found that the inhibitory effect of leptin on ERS may be related to the PI3K/Akt signaling pathway.

CIRI is a complex pathophysiological process that is regulated by a variety of factors, including ERS. The ER acts as a multifunctional organelle containing a multitude of ER chaperones, enzymes, and cofactors that regulate the precise folding of nascent proteins and assist polypeptides in obtaining their final functional conformations [28]. In the physiological environment, the protein folding efficiency of the ER is balanced with the rate of mRNA translation, and the ER membrane proteins PERK, IRE1, and ATF6 are inactivated by the binding of glucose-regulated protein 78 (GRP78) [29–31]. To a certain extent, ERS is an adaptive response of damaged cells that helps to restore cellular homeostasis, but excessive and persistent ERS activates proapoptotic signaling proteins such as cysteinyl aspartate specific proteinase 12 (Caspase12) and C/EBP homologous protein (CHOP), leading to apoptosis, which then aggravates CIRI. Under ischemic conditions, misfolded or unfolded proteins remain in the ER lumen and competitively bind GRP78, which results in the autophosphorylation of PERK and IRE1, the cleavage of ATF6 and thus the initiation of the UPR [32–34]. Extensive studies have revealed that ERS induced by CIRI may be a key factor in the pathological mechanism involving endothelial cell, glial cell and neuron loss after cerebral ischemia, and various *in vivo* and *in vitro* experiments have demonstrated that targeting the inhibition of ERS can effectively mitigate experimental CIRI [31,35,36]. The results of the present study showed that the expression of GRP78, p-PERK, p-IRE1 and ATF6 was significantly increased after MCAO/R, which was consistent with previous studies, suggesting that the PERK, IRE1 and ATF6 signaling pathways were activated during CIRI. In a pathological state, an abnormal ER ultrastructure is associated with ER function and signal transduction. TEM revealed that in damaged cortical neurons, the ER was swollen and partly fragmented, unlike its counterpart in normal cortical neurons, indicating that intense ERS in the *in vitro* model of CIRI might have shifted the UPR from early adaptive protective mechanism to programmed cell death. The activation of Caspase12 and the induction of CHOP expression are thought to be two specific apoptotic signaling pathways that occur during prolonged ERS [37,38]. Activated Caspase12 activates downstream Caspase family members such as Caspase3 and induces DNA fragmentation, which then results in a series of complex reactions that ultimately lead to apoptosis [39]. CHOP is expressed at low levels under physiological conditions, but its expression is significantly increased when cells encounter intense stimulation, which also induces cell death by up-regulating the protein levels of the Bcl family and Caspase12 [40]. Therefore, in the present study, we measured the expression of proapoptotic factors induced by ERS, CHOP and Caspase12, and the protein levels of the downstream apoptotic-related factors cleaved Caspase3, Bax and Bcl2. The results showed a distinct increase in the expression of CHOP, Caspase12, Bax and Caspase3 after OGD/R or MCAO/R, while the protein levels of Bcl2 were significantly down-regulated, further indicating the occurrence of ERS-related apoptosis in damaged neurons.

Leptin is a peptide hormone that binds its receptors to exert pleiotropic effects on biological functions. Leptin plays a universal role in promoting survival and proliferation, which has been confirmed in a variety of tissue and cell types [41,42]. Accumulating evidence emphasizes that leptin protects mitochondria, increases anti-inflammatory factor levels, and reduces excitatory neurotransmitter levels and apoptotic protein levels [14,43]. The results of the current study revealed that leptin effectively reduced *in vivo* and *in vitro* experimental CIRI, which is consistent with previous studies. Many researchers have demonstrated that exogenous leptin has a protective effect against brain damage, but its underlying mechanisms remain to be explored. Previous studies have shown that enhanced ERS and the activation of UPR pathways play a pathogenic role in leptin resistance [44]; later, scholars reported that in non-small-cell lung cancer, leptin down-regulates the protein level of CHOP through the PERK and ATF6 signaling pathways and thus inhibits apoptosis induced by ERS, revealing that ERS plays an important role in the biological effects of leptin [45]. Therefore, we assumed that leptin exerts its neuroprotective effect by inhibiting ERS induced by CIRI to promote neuronal survival. The results of this study provided evidence that leptin treatment further increased the expression of GRP78, which is considered a prosurvival protein, during ERS while significantly reducing expression of the proapoptotic factors CHOP and Caspase12 by activating the PERK, IRE1 and ATF6 pathways. TEM showed that OGD/R disrupted the integrity of the neuronal morphology and induced ultrastructural abnormalities in the ER, whereas leptin pretreatment reduced pathological changes in cell morphology and the ER structure, suggesting that the neuroprotection offered by leptin may be attributed to the inhibition of ERS and increased cell survival. Upon ischemic damage, imbalance between proapoptotic and antiapoptotic proteins leads to activation of the caspase cascade, which eventually results in cell apoptosis. Studies have shown that in the penumbral area, inhibiting the apoptotic process may be a therapeutic target to improve secondary events during brain injury after IS [46,47]. Our findings revealed that treatment with leptin protected neurons from CIRI, which was related to its ability to suppress ERS, which plays an important role in neuronal apoptosis via activation of the CHOP and Caspase12 pathways in IS. Furthermore, the Bax/Bcl2 ratio and the expression of cleaved Caspase3 indicated that the administration of leptin facilitated the activity of Bcl2 and limited the expression of Bax and Caspase-3.

An extensive number of studies have highlighted the neuroprotective effect of leptin in antiapoptotic mechanisms, which are related to a variety of pathways, including the PI3K/Akt pathway [16]. Consistent with previous findings, the results of this study showed a significant increase in PI3K and p-Akt protein levels under ischemia/reperfusion conditions, which was further increased after treatment with leptin, suggesting that leptin activates the PI3K/Akt signaling pathway during ischemia/reperfusion. However, after blocking the PI3K/Akt pathway by using LY294002, the neuroprotective effect of leptin was significantly decreased, as demonstrated by the increased infarct volume, pathological changes and neurological deficit scores, suggesting that leptin probably alleviated CIRI by regulating the PI3K/Akt signaling pathway. Studies in recent years have revealed that the PI3K/Akt signaling pathway is closely related to ERS. Bi et al. found that the ERS marker GRP78 migrates to the cell surface and interacts with PI3K under ischemic conditions [48]. Liu et al. demonstrated that the GRP78 and PI3K/Akt signaling pathways have bidirectional regulation, which promotes the survival and growth of post-OGD/R neural stem cells [49]. Therefore, a PI3K inhibitor was used to explore the altered expression of ERS-associated proteins. Our results revealed a significant increase in the protein levels of the ERS markers p-IRE1, CHOP and Caspase12, as well as the downstream proapoptotic proteins Bax and Caspase3, in the LY+leptin group compared with their expression the leptin group and a clear decrease in GRP78 and Bcl2 protein levels, suggesting that leptin inhibits CIRI-induced ERS, which may be related to the PI3K/Akt signaling pathway.

Improved mitigation of CIRI is a challenge in the treatment of IS. Our study preliminarily verified that leptin attenuates ERS and ERS-related death during reperfusion and that the PI3K/Akt signaling pathway may participate in its suppression of ERS. However, considering the differences between clinical practice and animal models and because leptin exerts its effects on multiple tissues, it is necessary to measure the efficacy and safety of leptin in patients with CIRI through high-quality and multicenter prospective studies. Therefore, the specific neuroprotective mechanism of leptin needs to be studied in the future.

## Conclusion

In summary, this study reveals that CIRI induces ERS in damaged cells, whereas leptin attenuates ERS and ERS-related death during reperfusion, and that the PI3K/Akt signaling pathway may be related to its inhibitory effect on ERS. These findings further elucidate the neuroprotective mechanism of leptin and provide a good experimental and theoretical basis for its clinical application for the treatment of IS.

## Data Availability

All the data that supports this study are available from the corresponding author on reasonable request.

## Abbreviations

CIRI: cerebral ischemic/reperfusion injury
ERS: endoplasmic reticulum stress
GRP78: glucose-regulated protein 78
IS: ischemic stroke
MCAO/R: middle cerebral artery occlusion and reperfusion
OGD/R: oxygen-glucose deprivation and reoxygenation
TTC: triphenyltetrazolium chloride

## References

[1] G.B.D. (2017) 2016 DALYs and HALE Collaborators. Global, regional, and national disability-adjusted life-years (DALYs) for 333 diseases and injuries and healthy life expectancy (HALE) for 195 countries and territories, 1990-2016: a systematic analysis for the Global Burden of Disease Study 2016. Lancet 390, 1260–1344 10.1016/S0140-6736(17)32130-X 28919118 PMC5605707

[2] Campbell B.C.V., De Silva D.A., Macleod M.R. et al. (2019) Ischaemic stroke. Nat. Rev. Dis. Primers 5, 70 10.1038/s41572-019-0118-8 31601801

[3] Matsumoto S., Murozono M., Kanazawa M., Nara T., Ozawa T. and Watanabe Y. (2018) Edaravone and cyclosporine A as neuroprotective agents for acute ischemic stroke. Acute Med. Surg. 5, 213–221 10.1002/ams2.343 29988669 PMC6028804

[4] El Khashab I.H., Abdelsalam R.M., Elbrairy A.I. and Attia A.S. (2019) Chrysin attenuates global cerebral ischemic reperfusion injury via suppression of oxidative stress, inflammation and apoptosis. Biomed. Pharmacother. 112, 108619 10.1016/j.biopha.2019.108619 30797156

[5] Liu L., Chen M., Lin K. et al. (2021) TRPC6 Attenuates cortical astrocytic apoptosis and inflammation in cerebral ischemic/reperfusion injury. Front. Cell Dev. Biol. 8, 594283 10.3389/fcell.2020.594283 33604333 PMC7884618

[6] Roussel B.D., Kruppa A.J., Miranda E., Crowther D.C., Lomas D.A. and Marciniak S.J. (2013) Endoplasmic reticulum dysfunction in neurological disease. Lancet Neurol. 12, 105–118 10.1016/S1474-4422(12)70238-7 23237905

[7] Groenendyk J., Agellon L.B. and Michalak M. (2021) Calcium signaling and endoplasmic reticulum stress. Int. Rev. Cell Mol. Biol. 363, 1–20 10.1016/bs.ircmb.2021.03.003 34392927

[8] Di Conza G. and Ho P.C.E.R. (2020) Stress responses: an emerging modulator for innate immunity. Cells 9, 695, Published 2020 Mar 12 10.3390/cells9030695 32178254 PMC7140669

[9] Zhong C.J., Chen M.M., Lu M., Ding J.H., Du R.H. and Hu G. (2019) Astrocyte-specific deletion of Kir6.1/K-ATP channel aggravates cerebral ischemia/reperfusion injury through endoplasmic reticulum stress in mice. Exp. Neurol. 311, 225–233 10.1016/j.expneurol.2018.10.005 30315808

[10] Pan B., Sun J., Liu Z. et al. (2021) Longxuetongluo Capsule protects against cerebral ischemia/reperfusion injury through endoplasmic reticulum stress and MAPK-mediated mechanisms. J. Adv. Res. 33, 215–225 10.1016/j.jare.2021.01.016 34603791 PMC8463917

[11] Wang K., Wang T., Xu H., Zhong X. and Huang Z. (2021) Harpagide exerts a neuroprotective effect by inhibiting endoplasmic reticulum stress via SERCA following oxygen-glucose deprivation/reoxygenation injury. Neurosci. Lett. 753, 135874 10.1016/j.neulet.2021.135874 33812930

[12] Herrington W., Lacey B., Sherliker P., Armitage J. and Lewington S. (2016) Epidemiology of atherosclerosis and the potential to reduce the global burden of atherothrombotic disease. Circ. Res. 118, 535–546 10.1161/CIRCRESAHA.115.307611 26892956

[13] Zahn K., Linseisen J., Heier M. et al. (2018) Body fat distribution and risk of incident ischemic stroke in men and women aged 50 to 74 years from the general population. The KORA Augsburg cohort study. PLoS ONE 13, e0191630, Published 2018 Feb 5 10.1371/journal.pone.0191630 29401461 PMC5798769

[14] Tang B.L. (2008) Leptin as a neuroprotective agent. Biochem. Biophys. Res. Commun. 368, 181–185 10.1016/j.bbrc.2008.01.063 18222172

[15] Gairolla J., Kler R., Modi M. and Khurana D. (2017) Leptin and adiponectin: pathophysiological role and possible therapeutic target of inflammation in ischemic stroke. Rev. Neurosci. 28, 295–306 10.1515/revneuro-2016-0055 28121618

[16] Zhang W.F., Jin Y.C., Li X.M., Yang Z., Wang D. and Cui J.J. (2019) Protective effects of leptin against cerebral ischemia/reperfusion injury. Exp. Ther. Med. 17, 3282–3290 10.3892/etm.2019.7377 30988703 PMC6447799

[17] Avraham Y., Dayan M., Lassri V. et al. (2013) Delayed leptin administration after stroke induces neurogenesis and angiogenesis. J. Neurosci. Res. 91, 187–195 10.1002/jnr.23147 23152300

[18] Yook J.S., Rakwal R., Shibato J. et al. (2019) Leptin in hippocampus mediates benefits of mild exercise by an antioxidant on neurogenesis and memory. Proc. Natl. Acad. Sci. U.S.A. 116, 10988–10993 10.1073/pnas.1815197116 31085646 PMC6561194

[19] Calió M.L., Mosini A.C., Marinho D.S. et al. (2021) Leptin enhances adult neurogenesis and reduces pathological features in a transgenic mouse model of Alzheimer's disease. Neurobiol. Dis. 148, 105219 10.1016/j.nbd.2020.105219 33301880

[20] Cui W., Wang S., Wang Z., Wang Z., Sun C. and Zhang Y. (2017) Inhibition of PTEN attenuates endoplasmic reticulum stress and apoptosis via activation of PI3K/AKT pathway in Alzheimer’s disease. Neurochem. Res. 42, 3052–3060 10.1007/s11064-017-2338-1 28819903

[21] Bi X., Zhang G., Wang X. et al. (2018) Endoplasmic reticulum chaperone GRP78 protects heart from ischemia/reperfusion injury through Akt activation. Circ. Res. 122, 1545–1554 10.1161/CIRCRESAHA.117.312641 29669712 PMC5970094

[22] Shen D., Chen R., Zhang L. et al. (2019) Sulodexide attenuates endoplasmic reticulum stress induced by myocardial ischaemia/reperfusion by activating the PI3K/Akt pathway. J. Cell. Mol. Med. 23, 5063–5075 10.1111/jcmm.14367 31120192 PMC6653332

[23] Zhang J., Deng Z., Liao J. et al. (2013) Leptin attenuates cerebral ischemia injury through the promotion of energy metabolism via the PI3K/Akt pathway. J. Cereb. Blood Flow Metab. 33, 567–574 10.1038/jcbfm.2012.202 23299243 PMC3618393

[24] Longa E.Z., Weinstein P.R., Carlson S. and Cummins R. (1989) Reversible middle cerebral artery occlusion without craniectomy in rats. Stroke 20, 84–91 10.1161/01.STR.20.1.84 2643202

[25] Ashafaq M., Tabassum H. and Parvez S. (2017) Modulation of behavioral deficits and neurodegeneration by tannic acid in experimental stroke challenged Wistar rats. Mol. Neurobiol. 54, 5941–5951 10.1007/s12035-016-0096-8 27678149

[26] Zhang W., Jin Y., Wang D. and Cui J. (2020) Neuroprotective effects of leptin on cerebral ischemia through JAK2/STAT3/PGC-1-mediated mitochondrial function modulation. Brain Res. Bull. 156, 118–130 10.1016/j.brainresbull.2020.01.002 31935431

[27] Sui M., Xu D., Zhao W. et al. (2021) CIRBP promotes ferroptosis by interacting with ELAVL1 and activating ferritinophagy during renal ischaemia-reperfusion injury. J. Cell. Mol. Med. 25, 6203–6216 10.1111/jcmm.16567 34114349 PMC8256344

[28] Yang W. and Paschen W. (2016) Unfolded protein response in brain ischemia: a timely update. J. Cereb. Blood Flow Metab. 36, 2044–2050 10.1177/0271678X16674488 27733676 PMC5363674

[29] Calfon M., Zeng H., Urano F. et al. (2002) IRE1 couples endoplasmic reticulum load to secretory capacity by processing the XBP-1 mRNA. Nature 415, 92–96 10.1038/415092a 11780124

[30] Glembotski C.C., Rosarda J.D. and Wiseman R.L. (2019) Proteostasis and beyond: ATF6 in ischemic disease. Trends Mol. Med. 25, 538–550 10.1016/j.molmed.2019.03.005 31078432 PMC6592750

[31] Wang Y., Zhang J.H., Sheng J. and Shao A. (2019) Immunoreactive cells after cerebral ischemia. Front. Immunol. 10, 2781 10.3389/fimmu.2019.02781 31849964 PMC6902047

[32] Luo D., He Y., Zhang H. et al. (2008) AIP1 is critical in transducing IRE1-mediated endoplasmic reticulum stress response. J. Biol. Chem. 283, 11905–11912 10.1074/jbc.M710557200 18281285 PMC2335342

[33] Yu Z., Sheng H., Liu S. et al. (2017) Activation of the ATF6 branch of the unfolded protein response in neurons improves stroke outcome. J. Cereb. Blood Flow Metab. 37, 1069–1079 10.1177/0271678X16650218 27217380 PMC5363481

[34] Fei H., Xiang P., Luo W. et al. (2021) CTRP1 attenuates cerebral ischemia/reperfusion injury via the PERK signaling pathway. Front. Cell Dev. Biol. 9, 700854 10.3389/fcell.2021.700854 34422821 PMC8371340

[35] Rissanen A., Sivenius J. and Jolkkonen J. (2006) Prolonged bihemispheric alterations in unfolded protein response related gene expression after experimental stroke. Brain Res. 1087, 60–66 10.1016/j.brainres.2006.02.095 16684512

[36] Haupt M., Zechmeister B., Bosche B. et al. (2020) Lithium enhances post-stroke blood-brain barrier integrity, activates the MAPK/ERK1/2 pathway and alters immune cell migration in mice. Neuropharmacology 181, 108357 10.1016/j.neuropharm.2020.108357 33065166

[37] García de la Cadena S. and Massieu L. (2016) Caspases and their role in inflammation and ischemic neuronal death. Focus on caspase-12. Apoptosis 21, 763–777 10.1007/s10495-016-1247-0 27142195

[38] Hu H., Tian M., Ding C. and Yu S. (2019) The C/EBP homologous protein (CHOP) transcription factor functions in endoplasmic reticulum stress-induced apoptosis and microbial infection. Front. Immunol. 9, 3083 10.3389/fimmu.2018.03083 30662442 PMC6328441

[39] Shimoke K., Matsuki Y., Fukunaga K. et al. (2011) Appearance of nuclear-sorted caspase-12 fragments in cerebral cortical and hippocampal neurons in rats damaged by autologous blood clot embolic brain infarctions. Cell. Mol. Neurobiol. 31, 795–802 10.1007/s10571-011-9687-0 21476018 PMC11498496

[40] Su Y. and Li F. (2016) Endoplasmic reticulum stress in brain ischemia. Int. J. Neurosci. 126, 681–691 10.3109/00207454.2015.1059836 26289799

[41] McGregor G. and Harvey J. (2018) Regulation of hippocampal synaptic function by the metabolic hormone, leptin: implications for health and neurodegenerative disease. Front Cell Neurosci. 12, 340 10.3389/fncel.2018.00340 30386207 PMC6198461

[42] Ekraminasab S., Dolatshahi M., Sabahi M., Mardani M. and Rashedi S. (2022) The interactions between adipose tissue secretions and Parkinson's disease: The role of leptin. Eur. J. Neurosci. 55, 873–891 10.1111/ejn.15594 34989050

[43] Hu S., Cheng D., Peng D., Tan J., Huang Y. and Chen C. (2019) Leptin attenuates cerebral ischemic injury in rats by modulating the mitochondrial electron transport chain via the mitochondrial STAT3 pathway. Brain Behav. 9, e01200 10.1002/brb3.1200 30632310 PMC6379515

[44] Ye Z., Liu G., Guo J. and Su Z. (2018) Hypothalamic endoplasmic reticulum stress as a key mediator of obesity-induced leptin resistance. Obes. Rev. 19, 770–785 10.1111/obr.12673 29514392

[45] Lai Q. and Sun Y. (2013) Human leptin protein induces proliferation of A549 cells via inhibition of PKR-like ER kinase and activating transcription factor-6 mediated apoptosis. Yonsei Med. J. 54, 1407–1415 10.3349/ymj.2013.54.6.1407 24142645 PMC3809871

[46] Tabas I. and Ron D. (2011) Integrating the mechanisms of apoptosis induced by endoplasmic reticulum stress. Nat. Cell Biol. 13, 184–190 10.1038/ncb0311-184 21364565 PMC3107571

[47] Uzdensky A.B. (2019) Apoptosis regulation in the penumbra after ischemic stroke: expression of pro- and antiapoptotic proteins. Apoptosis 24, 687–702 10.1007/s10495-019-01556-6 31256300

[48] Bi X., Zhang G., Wang X. et al. (2018) Endoplasmic reticulum chaperone GRP78 protects heart from ischemia/reperfusion injury through Akt activation. Circ. Res. 122, 1545–1554 10.1161/CIRCRESAHA.117.312641 29669712 PMC5970094

[49] Liu Q., Li Y., Zhou L. et al. (2018) GRP78 promotes neural stem cell antiapoptosis and survival in response to oxygen-glucose deprivation (OGD)/reoxygenation through PI3K/Akt, ERK1/2, and NF-κB/p65 pathways. Oxid. Med. Cell Longev. 2018, 3541807, Published 2018 Apr 10 10.1155/2018/3541807 29849883 PMC5914129

